# Antimicrobial Resistance—Theory and Methods

**DOI:** 10.1093/jacamr/dlz008

**Published:** 2019-04-12

## Abstract

**Graphical Abstract:**
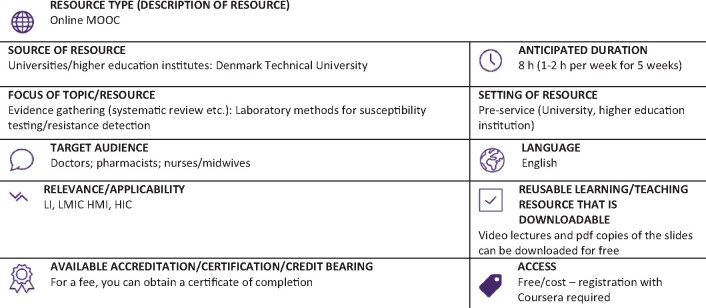

**Resource web link:** **https://www.coursera.org/learn/antimicrobial-resistance** (Full classification scheme available at: http://bsac.org.uk/wp-content/uploads/2019/03/Educational-resource-review-classification-scheme.pdf)

**WHO region and country (World Bank):** Europe, Denmark (HIC)

## Peer review commentary

This is an online course consisting of six modules (a series of videos of PowerPoint presentations) focusing on antimicrobials and their mode of action, antimicrobial resistance mechanisms (with a specific focus on MRSA, ESBL/AmpC/carbapenemase-producing Gram-negatives and colistin resistance), antimicrobial susceptibility testing methodology, quality assurance methods for laboratory testing and WGS for detecting resistance. Most modules have an in-video quiz and there is another quiz at the end of each module that is graded and counts towards the learner’s final grade. Feedback on the correct answers is provided once the quiz has been completed.

This resource is primarily aimed at laboratory staff (but will also be of interest to doctors, pharmacists, nurses and healthcare students) who need to understand the basics of antimicrobial resistance and methods to determine susceptibility of organisms to antibiotics and the quality control/quality assurance aspects relating to running a service that provides susceptibility testing. It might be useful to others who want to understand resistance mechanisms and how antimicrobial susceptibility testing is done, but there are probably more relevant resources out there that provide the information more concisely. The end-of-module quizzes are a good way to test if the viewers have retained the information, but the wording of some of these can be a little confusing. One strength of the course is that new modules have been added since it was first launched, covering resistance in Gram-negative organisms and MRSA in more detail.

